# Data- and expert-driven rule induction and filtering framework for functional interpretation and description of gene sets

**DOI:** 10.1186/s13326-017-0129-x

**Published:** 2017-06-26

**Authors:** Aleksandra Gruca, Marek Sikora

**Affiliations:** Institute of Informatics, Silesian University of Technology, Akademicka 16, Gliwice, 44-100 Poland

**Keywords:** Functional description, Gene Ontology, Logical rules, Expert-driven rule induction

## Abstract

**Background:**

High-throughput methods in molecular biology provided researchers with abundance of experimental data that need to be interpreted in order to understand the experimental results. Manual methods of functional gene/protein group interpretation are expensive and time-consuming; therefore, there is a need to develop new efficient data mining methods and bioinformatics tools that could support the expert in the process of functional analysis of experimental results.

**Results:**

In this study, we propose a comprehensive framework for the induction of logical rules in the form of combinations of Gene Ontology (GO) terms for functional interpretation of gene sets. Within the framework, we present four approaches: the fully automated method of rule induction without filtering, rule induction method with filtering, expert-driven rule filtering method based on additive utility functions, and expert-driven rule induction method based on the so-called seed or expert terms – the GO terms of special interest which should be included into the description. These GO terms usually describe some processes or pathways of particular interest, which are related to the experiment that is being performed. During the rule induction and filtering processes such seed terms are used as a base on which the description is build.

**Conclusion:**

We compare the descriptions obtained with different algorithms of rule induction and filtering and show that a filtering step is required to reduce the number of rules in the output set so that they could be analyzed by a human expert. However, filtering may remove information from the output rule set which is potentially interesting for the expert. Therefore, in the study, we present two methods that involve interaction with the expert during the process of rule induction. Both of them are able to reduce the number of rules, but only in the case of the method based on seed terms, each of the created rule includes expert terms in combination with the other terms. Further analysis of such combinations may provide new knowledge about biological processes and their combination with other pathways related to genes described by the rules. A suite of Matlab scripts that provide the functionality of a comprehensive framework for the rule induction and filtering presented in this study is available free of charge at: http://rulego.polsl.pl/framework.

**Electronic supplementary material:**

The online version of this article (doi:10.1186/s13326-017-0129-x) contains supplementary material, which is available to authorized users.

## Background

### Introdution

Over 20 years ago, high-throughput technologies for the analysis of genomic data opened a new era in molecular biology and genetics. Since the beginning of the so-called genomic era, advanced tools and techniques such as DNA microarrays [1] and next-generation sequencing (NGS) [2] systems allow for studying genomes, analyzing cellular processes and interactions, which is the first step of research leading to diagnosis of diseases and invention of new drug, and treatment discovery [3–5]. However, to be effective, today’s genomic technologies require not only reagents and sophisticated laboratory instruments but also application of new software, algorithms, and knowledge discovery techniques in order to process and analyze huge amount of experimental data [6–8].

Many of the experiments using genomic technologies are focused on searching of co-regulated genes that play an important role in some biological processes particularly interesting from the experimental point of view. Typically, genes that work coordinately as gene modules or gene networks are seen as groups characterized by similar expression levels and can be found by applying clustering methods to the expression data [9–13]. However, the functional analysis and interpretation of gene clusters obtained in such a way are difficult and time-consuming, especially if each gene composing the group is manually analyzed by an expert in the field, based on his or her experience and literature searches.

To help the expert during such analysis, a lot of tools have been invented and successfully applied during last years. One of the most frequently used tools is the Gene Ontology (GO) database, which is a collaborative effort to address the need for consistent descriptions of gene products across databases [14]. The information in the GO database is divided into three separate structures in the form of directed acyclic graphs (DAGs): Biological Process (BP), Molecular Function (MF) and Cellular Component (CC). Each node of the graph has a label *t* called the Gene Ontology term and has a unique seven-digit number, name, short description, and defined relationship to one or more terms in the same domain.

The information included in the GO database is provided on different levels of specificity: the terms found closer to the root of the graph (higher in the hierarchy) are general descriptions, and as the graph is traversed down to its leaves, the terms become more and more specific. The important part of GO database are annotations that associate gene products with particular terms in Gene Ontology graph. Each gene product can be annotated to zero or more terms of any ontology on any level of the GO graph. Annotations are independent of each other, but should be made on the most detailed level in the ontology as annotating to a particular term implies annotation to all its parent terms up to the root.

In this paper, we describe a comprehensive framework for functional description of gene sets based on the so-called logical rules that are combinations of GO terms. The presented approach involves (i) method of rule induction which takes into account the structure of Gene Ontology database, (ii) method of rule interestingness assessment based on various subjective and objective criteria, and (iii) the method of rule filtering that allows removing the rules that are uninteresting from the expert point of view from the output rule set. Finally, (iv) we present a new, semi-interactive method of rule induction which allows the expert to influence the process of rule generation by providing a set of so-called seed or expert terms, that is the GO terms of special interest, which should be included into the description. These GO terms usually describe some processes of particular interest, frequently related to the experiment that is being performed. During the rule induction and filtering process such seed terms are used as a base on which the description is built.

### Using Gene Ontology database for functional analysis

The first approach to the automated functional interpretation was the so-called single-term analysis in which, based on the results of the statistical test, a list of over-represented GO terms describing gene groups was obtained. A number of tools were created based on the idea of single-term analysis, which is still the most common approach used for functional interpretation of gene sets [15].

Another approach to the methods of automated functional interpretation was the introduction of more advanced tools such as RuleGO [16] or GeneCodis [17] that search for the so-called logical rules that include combinations of GO terms. The rationale standing behind such approach is that the combinations of GO terms are more specific and therefore can show significance, whereas single terms do not show statistically significant enrichment or depletion. If we analyze GO terms separately, some of them may be too general to be included in the list of statically significant terms; however, their combination with other terms may present some novel and interesting information.

In our previous research [18], we showed that the number of possible statistically significant combinations of co-existing pathways is huge and that a filtering step is required in order to reduce the number of possible results. However, frequently, an expert who designs an experiment might be interested in some specific process or event related to the research. For example, in cancer research searching for a gene signature, which could be potentially useful for diagnosis or could suggest novel drug targets, one may look for genes involved in particular biological process or network related to transformation of normal cells into cancer cells. Therefore, there is a concern that automated filtering methods could remove some rules that consists of GO terms potentially interesting to the expert. To address this issue, we propose a new methodology of rule induction and filtering which allows for including the expert domain knowledge into rule generation and filtering process. The new approach is based on the RuleGO algorithm, and it allows the expert to influence the process of rule generation by defining the GO terms of special interests, which are then included into the rules and preserved in the output rule set after the filtering step.

### Related work

So far, to find co-appearance of Gene Ontology terms, association rule induction algorithms were applied. Caramona-Saez et al. [19] proposed a method that combines expression data and biological information. Later, in another study, Caramona-Saez et al. [20] introduced the Genecodis web-based tool for integrated analysis of annotations from different sources. The method uses the Apriori algorithm [21] to discover sets of annotations that frequently co–occur in the analyzed group of genes. A similar tool that allows finding combinations of annotations from many different sources such as functional categories, gene regulation, sequence properties, evolution, and conservation was presented by Hackenberg et al. [22]. Also, Gruca [23] applied FP-growth algorithm to find combinations of GO terms for functional description of genes.

Research on the induction of rules that combine gene expression data and biological information was also performed [24–26]. For example, in Lopez et al. [25], gene groups described by similar values of the so-called structural features (e.g., gene length, the number of nucleotides in the coding sequence, gene G+C content) with the corresponding GO terms are also joined by means of association rules. Hvidsten et al. [27] proposed conditional rules of the form "IF conjunction of conditions describing time series of gene expression profile THEN GO term". In a rule conclusion, a set of Gene Ontology terms describing the group were included.

Rule induction techniques mentioned earlier have two basic drawbacks that can make obtained rules difficult or even impossible to interpret. First, known rule induction methods do not consider the fact that hierarchy of GO terms could result in replacing a conjunction of attributes with one, more specific GO term at the lowest level in the GO graph hierarchy. Second, all the methods mentioned earlier lead to generate a huge number of rules without providing more advanced (apart from a *p*-value and a rule coverage) methods of rule interestingness evaluation and rule filtering.

In a previous study [18], we proposed the rule induction algorithm which takes into account the structure of the Gene Ontology graph and the method of selection of the most important GO terms. The selection method is based on the Rough Set Theory [28] and the asymmetrical indiscernibility relation. However, the number of induced rules was still too large. Therefore, another method for rule filtering based on subjective rule attractiveness measure was proposed in Gruca and Sikora [29].

The problem of finding the minimal subset of the set of rules, which has lower complexity and simultaneously maximizes the value of the specified criterion (e.g., overall classification accuracy) is NP-complete and computationally expensive. For descriptive purpose or when the classification ability is not the most important feature, the rule elimination procedures (rule filtering) are based on the minimum interestingness requirements (typically some well-known rule interestingness measures are chosen) [30, 31]. Some papers also refer to multicriteria rule evaluation, and in such a case, machine learning [32] and multicriteria decision-making [33] methods are applied. These methods can be called supervised because they use information obtained from an expert. For example, Lenca [33] apply the PROMETHEE method [34] to select interestingness measure which is able to order a rule set in a manner most similar to the order provided by an expert.

In biological or medical applications, it is very important to determine the rules containing information that is interesting for a user. However, automatic selection of elementary conditions included in the rule premises is the main principle of rule induction algorithms, and rules induced in this way may not always include knowledge that is interesting and useful to the user.

To date, few studies have described how to design the induction algorithm in such a way that it takes into account the user preferences. Stefanowski and Vanderpooten [31] present the Explore algorithm, which is based on the idea of the Apriori method and allows the user to specify the requirements for attributes and/or their values, appearing in the rule premises.

Other papers on the induction of association rules describe examples of interactive construction of rules [35] and the induction of the so-called unexpected rules. Unexpected rules are created on the basis of user-defined templates, indicating the attributes included in the so-called typical rules [36]. Gamberger and Lavrac [37] present a similar proposal for the decision rule induction algorithm, intended for descriptive purposes.

Algorithms using the paradigm of argument-based learning [38, 39] allow the user to provide explanation for each example as to why it has been classified into that particular decision class. Examples of medical applications show that this approach can significantly reduce the set of generated rules. However, the argument-based learning approach does not verify the hypotheses that represent the dependencies that, in the user opinion, might occur in the data. Partially, this possibility is presented in Chen and Liu [40], where the user defines a set of rules that he or she expects to find in the analyzed dataset. Then the rule-based version of the C4.5 algorithm is executed, and three types of rules are generated: consistent with the rules defined by the user, not related to the user rules, and inconsistent with the user knowledge. The rule *r* is considered to be consistent with the user knowledge if, in the set of defined rules, there is at least one rule *e* such that *r* and *e* indicate the same decision class and a set of examples covered by *r* is a subset of examples covered by *e*.

## Methods

### Rule induction

Let us assume that there are two sets of genes: *G*_1_ which is a set that we want to describe functionally and *G*_2_ which is a reference set, and *G*={*G*_1_∪*G*_2_}. Also there is a set of GO terms *T* describing genes and gene products. Formally, Gene Ontology is a directed acyclic graph denoted as *G* *O*=(*T*,≤), where ≤ is a binary relation on *T* such that genes described by the GO term *t*_*j*_∈*T* are a subset of genes described by the GO term *t*_*i*_∈*T*, where *t*_*j*_≤*t*_*i*_, if and only if there exists a path (*t*_*i*_,*t*_*i*+1_,...,*t*_*j*−1_,*t*_*j*_) such that *t*_*m*_≤*t*_*m*−1_ for *m*=*i*+1,*i*+2,..,*j*−1,*j*. The largest element *t*_0_ is a root of DAG and the *i*-th level of the graph is formed by all the GO terms *t*∈*T* for which there is a path (*r* *o* *o* *t*,*t*_1_,...*t*_*i*−1_,*t*_*i*_) such that *t*_1_≤*r* *o* *o* *t*,*t*_*m*_≤*t*_*m*−1_ for *m*=2,3,...,*i*−1 and *t*_*i*_≤*t*_*i*−1_.

Each gene *g* from the set *G* can be described (annotated) by a one or more Gene Ontology terms at any level of GO graph. Therefore, it is possible to create a so-called decision table **D** **T**=(*G*,*T*∪{*d*}), where for all *t*∈*T*,*t*:*G*→{0,1} and *d*(*g*)∈{*G*_1_,*G*_2_} for all *g*∈*G*. Each row in **D** **T** represents a description of a single gene *g*∈*G* by the GO terms, annotating it from the set *T*. The notation *t*(*g*)=1 (called a positive descriptor) denotes that a gene *g* is annotated by the term *t*, whereas *t*(*g*)=0 (called a negative descriptor) means the opposite. Each gene is also assigned to one of the groups *G*_1_ or *G*_2_. The task is to find all statistically significant logical rules (combinations of GO terms) of the following form:

1 $$ r : \text{IF}~t_{i1}~\text{{and}}~{t_{i2}}~\text{{and}}~...~ \text{{and}}~{t_{ik}}~\text{{THEN}}~{G_{1}},  $$

where *t*_1_,*t*_2_,...,*t*_*i*_⊆*T*. The interpretation of the above logical rule is as follows: *if a gene is described by a conjunction of Gene Ontology terms appearing in the rule premise, then it belongs to a group of genes indicated in the rule conclusion*. The set of rules creates functional description of the gene group *G*_1_. In order to simplify the notation, we include only positive descriptors into the rule premise.

In our case, the generation of the rules is discovery oriented. Therefore, we search for all co-occurring combinations of GO terms satisfying some criteria defined by the user. Such approach is, among others, implemented in the classical association rule induction algorithm Apriori [21] and its extension for decision rule induction, Explore [31].

The aim of the method is to generate all statistically significant logical rules of defined length, with premises containing only positive descriptors. The induced rules have to satisfy some additional criteria defined by a user (e.g., a minimum number of genes describing each of induced rules).

Therefore, to create the description of the given group, we must determine all possible combinations of GO terms describing that group. In pessimistic case (assuming that every generated combination of GO terms is statistically significant), this would result in the following number of generated rules $\sum _{k=1}^{|T|}\binom {|T|}{k}=2^{|T|}-1$, where |*T*| is the number of all GO terms considered.

In order to narrow down the searching space and shorten the algorithm operating time, we introduced several modifications to the Explore algorithm [18]. The basic method is based on the idea of the so-called rule candidates. The generation of rules starts from a single GO term, and then, in the loop, the rule is extended by adding another GO term. Proposed solution assures that all GO terms that are included in the rule premise belong to exclusive paths leading from these terms to the root. In other words, there is no such GO term in the rule premise that is in the relation ≤ with any other GO term from the rule premise, which means that among all GO terms that create the premise of a single rule, there are no such two GO terms that are in parent-child relationship, according to the structure of DAG.

We say that a gene is recognized by the rule if it is described by all GO terms from the rule premise and that a gene is supported by the rule, if it is recognized by the rule and belongs to the group indicated in the rule conclusion.

Below, we present pseudocodes of procedures that allow to generate rules. The *Eliminate* procedure removes terms that are placed too close to the root in the GO ontology graph from the GO terms set. In particular, the user does not need to define any restrictions. In such a case, the *Eliminate* procedure returns a set of terms that are identical to the submitted ones.







The rule *r* which is statistically significant (the first condition *if* in the *GoodCandidates* procedure) is added to the output set of rules. However, it is not removed from the set of candidate rules because its extension may lead to obtain another, successive, statistically significant rule. The candidate rule is removed from the *Lr* list if it does not fulfill the minimal support criterion (*minSupp*). The value of *minSupp* is defined by the user as an algorithm parameter.

Our previous research [18] revealed that the number of generated rules that are statistically significant combinations of GO terms is usually very big.

Typically, even when describing small datasets consisting of several hundreds of genes, the outcome number of statistically significant rules can be around several hundred thousands. Therefore, sophisticated filtering methods must be applied before presenting the results to the expert. In the following subsections, we present several possible filtering approaches.

#### Rule interestingness

As mentioned in the previous section the rules are generated for the description purposes. We would like to stress that it is very difficult or even impossible to provide the definition of the *interesting* rule. Depending on the expertise of the person who performs the experiment and the purpose of analyses, different aspects of the description might be important. In general, the criteria on which individual rule is evaluated might be objective or subjective [30]. For each rule, we can determine *p* – number of positive examples, that is, number of genes from *G*_1_ described by this rule, *n* – number of negative examples, that is, number of genes from *G*_2_ described by this rule. *P* denotes all positive examples, that is, genes belonging to *G*_1_, and *N* denotes genes belonging to *G*_2_. A lot of measures have been defined in the literature based on the values of *p*,*P*,*n* and *N* [41–44].

Two most basic measures that can be used to assess rule quality are precision: *p* *r* *e* *c*(*r*)=*p*/(*p*+*n*) and coverage: *c* *o* *v*=*p*/*P*. The first measure describes how likely the rule is able to describe examples from the positive set. The second one describes how general is the rule, that is, the percentage of genes from the positive set that are described (covered) by the rule. Typically, we search for the rules that are characterized by both high precision and coverage. Therefore, in the literature, a lot of measures have been defined, which combine both precision and coverage in one single, more powerful measure that represents trade-off between these two elements.

One of such examples is Correlation measure *Corr* that is used in the FOSSIL rule induction algorithm and for association rules evaluation [43] and is computed as follows:

2 $$ Corr(r)=\frac{pN-Pn}{\sqrt{PN(p+n)(P-p+N-n)}}.  $$

The *Corr* measure takes into account the number of positive and negative examples described by the rule and also analyzes additional information about the dependencies between *p*, *n*, *P*, and *N*. This is extremely useful while generating rules for classification purposes. However, in case the rules are generated for description, we also need to evaluate the rules by using some other additional criteria, not only the rule ability to discriminate between positive and negative examples.

In the presented framework, for the rule interestingness assessment, we propose to use *Q* *C* *o* *m* *p* *o* *u* *n* *d* interestingness measure. This approach was introduced in our previous RuleGO method [16]. The *Q* *C* *o* *m* *p* *o* *u* *n* *d* measure is a product of several rule quality measures and is computed as follows:

3 $$ {} QCompound(r)=Lenght(r)\ast GO\_Depth(r)\ast mY\: AILS(r),  $$

where *L* *e* *n* *g* *t* *h*(*r*) represents a number of GO terms in the rule premise (the longer is the rule, the better as it includes more knowledge), *G* *O*_*D* *e* *p* *t* *h*(*r*) is a normalized sum of levels in GO graph of the terms from rule premise and *m* *Y* *A* *I* *L* *S*(*r*)=(0.5+0.25*p* *r* *e* *c*(*r*))*p* *r* *e* *c*(*r*)+(0.5−0.25*c* *o* *v*(*r*))*c* *o* *v*(*r*) is modified YAILS measure [45] that evaluates both rule precision and coverage. Therefore, the proposed measure takes into account not only classification abilities of the rule but also the structure of the information included in the rule premise. In the framework presented in this paper, the user can customize the rule interestingness measure by including or excluding particular components from it, which allows for evaluating different aspects of the rule quality.

In addition to the typical quality or interestingness measures that are used for rule quality assessment in the field of data mining, in the study, we also perform over-representation test to determine statistical significance of the rules. This is a typical approach that is used in many single-term gene enrichment systems for the functional analysis, and the most commonly used statistic for evaluating which functional categories are enriched in a set of genes is hypergeomteric test (as presented in Table 2 in [15]). Hypergeomteric test analyzes enrichment by evaluating the ratio of genes described by the rule in the analyzed gene set *G*_1_ to genes described by the rule in the reference set *G*_2_. Genes in the analyzed gene set are assumed to have an equal likelihood of being identified, consistent with the null model of hypergeomteric test.

The rule generation method presented in this study assumes that we add only the rules that are statistically significant to the output set; therefore, for each rule, we compute its *p*-value according to the hypergeometric test. As we perform enrichment analysis, that is as we search the combinations of GO terms that are overrepresented in the analyzed gene set, in our analysis, we use the right-sided hypergeometric test. To correct for multiple testing, we provide corrected *p*-value according to Benjamini and Hochberg procedure to control False Discovery Rate [46].

#### Filtering and selecting the most relevant rules

Filtering is the process of selection of the most important/interesting rules from the whole set of generated rules. Most filtering methods are based on rule quality rankings. The schema of the simplest filtering procedure is as follows: first, each rule is evaluated according to the arbitrarily selected rule interestingness measure; then, the ranking of the rules is created, and, in the last step, based on the ranking (and some additional criteria if applicable), the rules of the lower quality are removed from the output rule set.

It is important that during the rule removal process, the filtering procedure must also take into account the coverage (number of genes described by the rules) of the described set of genes. The method should be designed in such a manner that the coverage of analyzed gene group is the same before and after filtering.

In the presented framework, after computing the *Q* *C* *o* *m* *p* *o* *u* *n* *d* interestingness measure for each rule, the rule set is ordered according to its value. Then, based on the ranking, the two-step filtering procedure is performed. In the first step, for each rule, the method analyzes whether another rule, lower in the ranking, exists, which supports the same set of genes (or its subset). In such a case, that rule is a candidate to be removed from the output rule set. However, before removal of any rule, its similarity to the rule which is higher in the ranking is analyzed. This is because rules are generated for description purposes and removal of any rule from the output rule set may result in the removal of potentially interesting information. Therefore, the dissimilarity measure analyzes the GO composition of premises of two rules *r*_*j*_ and *r*_*j*_ in the following way:

4 $$ {} sim(r_{i},r_{j})=1-\frac{\sharp uGOterms(r_{i},r_{j})+\sharp uGOterms(r_{j},r_{i})}{\sharp GOterms(r_{i})+\sharp GOterms(r_{j})},  $$

where *♯* *u* *G* *O* *t* *e* *r* *m* *s*(*r*_*i*_,*r*_*j*_) is the number of unique GO terms occurring in the premise of the rule *r*_*i*_ and not occurring in the premise of the rule *r*_*j*_, and *♯* *G* *O* *t* *e* *r* *m* *s*(*r*_*i*_) and *♯* *G* *O* *t* *e* *r* *m* *s*(*r*_*j*_) denote the number of GO terms in the premises of the rules *r*_*i*_ and *r*_*j*_, respectively. We assume that a GO term *t* from *r*_*i*_ is unique if there is no parent-child or child-parent relationship of that term with any of the GO terms from *r*_*j*_ premise.

If two rules are dissimilar above the defined dissimilarity measure threshold, then both of them will remain in the output rule set. Usually, after this step, the number of rules is still large; therefore, the user has the possibility to apply the second part of the filtering process.

The second step of the filtering procedure is also based on the rule dissimilarity analysis. In this part of the method, we traverse the rule ranking from top to the bottom and analyze the dissimilarity among the rules. In the output rule set, we leave only such rules that are dissimilar to each other above the defined threshold. The rationale standing behind such approach is that, in the description, we want to include only the most distinct processes. However, as already mentioned, we also do not want to reduce the coverage of the resulting rule set by removing too much rules from the description. Therefore, if removal of the rule *r* would change the rule set coverage (i.e., there are no other rules left in the description supporting the same set of genes as rule *r* supports), the rule remains in the output rule set. The procedure of the filtering process (first step) is presented in Algorithm 4.



Method of rule set generation and its filtering presented above is a fully automated approach to the rule induction for description purposes. The expert may influence the filtering process, by customizing the *Q* *C* *o* *m* *p* *o* *u* *n* *d* measure that evaluates the rule interestingness or by defining if filtering process should have one or two steps, depending on the number of rules in the output dataset. However, most of this process is carried out in an automated way, and hence, there is a risk that some combinations of pathways that could be interesting from the expert’s point of view are removed from the output dataset.

#### Expert-driven rule evaluation by UTA method

In our framework, we propose another approach that allows generating rules that are more consistent with the expert preferences [29]. Here, the user is presented with a small set of selected rules that should be representative for the whole dataset. The rules presented to the user are selected in the following way: first, each generated rule is evaluated with several rule interestingness measures, and for each measure, we can determine its minimal and maximal values which give us the range of possible values for this measure. Then, the range of each partial measure is divided into three intervals, and one representative rule is randomly selected from each interval.

In the proposed framework, for each rule, five partial rule quality measures *q*_*i*_(*r*) are defined. These measures asses the quality of the rules from both subjective and objective points of view. In particular, for each rule, the following quality indicies are evaluated: *mYAILS*, *Corr*, *p*−*v* *a* *l* *u* *e*, *length*, and *G* *O*_*D* *e* *p* *t* *h*. The first two measures take into account the composition of genes in positive and negative sets, third measure is based on hypergeometric statistical test, and all of them could be regarded as objective measures. Other two measures are more oriented for the description as they are focused on the structure of information included in the rule premise.

In the framework proposed in this study, the expert is presented with 15 representative rules and they are ranked in preferred, subjective order – the most interesting rule from the user point of view is placed on the top of the ranking, the less interesting one goes to the bottom. The order provided by the expert is used to generate the so-called partial utility functions *u*_*i*_ that are used to estimate additive utility function (UTA measure) [47]. The partial utility functions (especially estimation of *u*_*i*_ and *w*_*i*_) are computed in such a way that the ranking of the rules based on the UTA measure reflects the ranking defined by the expert. The *Q*_*UTA*_ measure is computed as follows:

5 $$ Q_{UTA}=u_{i}(q_{i}(r))w_{i},  $$

where *r* denotes the evaluated rule, *q*_*i*_ is *i*-th rule quality measure, *u*_*i*_ is the estimated partial utility function measure, *w*_*i*_ is *i*-th coefficient and *i*=1,2,…,5.

In the next step, the *Q*_*UTA*_ measure is used to order all the rules from the output rule set, the standard two-step filtering procedure is applied, and the final, reduced output rule set is generated. Detailed description of the method can be found in Gruca and Sikora [29].

#### Expert-driven rule induction

In the rule induction and filtering methods discussed above, the user does not have any influence on the process of selection of attributes that are used by the rule induction algorithm. It is not difficult to imagine the situation when rules with the attributes describing particular process or pathway related to the experiment and therefore interesting from the user point of view are removed from the output rule set during the filtering step.

For example, in the research presented in this study, the set of rules, generated by RuleGO algorithm without filtering, includes 3,812 combinations, and after two-step filtering process, the number of rules is reduced to 32. The coverage (that is, number of genes described by the logical rules) remains the same, but a lot of possibly important information is removed from the resulting description.

To address this problem, we present here the extension of the rule induction algorithm that allows the user to influence the rule generation process by providing the list of pathways/processes of special interest that should be included in the rules composing the final result set. Such list of processes may be understood as a definition of a particular hypothesis on the function of genes composing the group that needs to be verified by the expert. The method assures that each rule from the output set includes one or more pathways from the set defined by the user, assuming that these pathways are functionally related to the gene group, that is, they annotate some genes from the group.

The user submits a list of GO terms, so-called seed or expert terms that are a base for the rule induction algorithm. The method analyzes the hierarchical structure of the GO graph and extends the set of seed terms with all their child processes. Then, the set of seed terms is filtered to the terms that satisfies the constrains defined by the user such as minimal and maximal depth in GO graph, minimal number of GO terms describing genes, evidence code, and so on. Next, all the seed terms create singe-element rules that are a base for the the logical rules generated by modified Explore algorithm described in *Rule induction* section.

With every iteration of the algorithm, the rules are built in such a way that they satisfy the following user requirements:

- each generated rule includes at least one GO term from the set of seed terms, extended by the child terms derived from the analysis of the GO graph hierarchy (if such seed terms describe genes from analyzed gene set)
- each generated rule is statistically significant (significance level is provided by the user)
- each generated rule includes GO terms satisfying user preferences concerning minimal number of genes described by a single GO terms and minimal rule support (parameters provided by the user).

After the rule set is generated, for each rule its interestingness is computed and based on this, the the rules are ranked accordingly. Then, if the output set includes a large number of rules, the user can apply one- or two-step filtering procedure as implemented in the standard RuleGO algorithm.

#### The comprehensive framework for functional description of gene sets

In the earlier sections, we presented several different approaches to the induction of logical rules. In this study, we would like to propose all those approaches as the elements of a bigger system which is a comprehensive framework for the induction of rules for description purposes. Depending on the aims and expertise of the person who performs the analyses, different steps can be applied in order to obtain the best functional description of the gene set.

First, the expert needs to make a decision whether the rule induction process should be automated or expert-driven. If there is no specific hypothesis related to the experiment, we suggest selection of automated rule induction. However, if the expert performing analyses is interested in particular processes and wants to see if there are cross talks among pathways of special interests and other pathways, then the expert-driven method of rule induction should be performed. In both the cases, the user has to set up the parameters for GO terms used to annotate genes and for induced rules. In case of GO terms, the user has to define which aspect of Gene Ontology should be taken into account (Biological Process, Molecular Function, Cellular Component), minimal number of genes that are described by GO term, minimal and maximal level of a GO term in GO graph, if GO terms with IEA evidence code should be excluded from the analysis, and if hierarchy of GO graph should be taken into account during the annotation process of GO terms. For the rule induction algorithm part, the user can define statistical significance threshold, maximal numbers of GO terms in a rule, minimal support, and maximal number of generated rules.

**Fig. 1:**
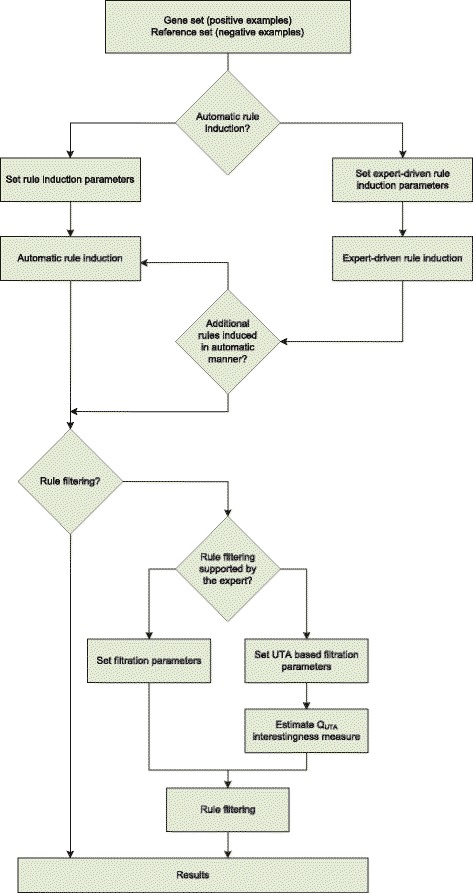
The workflow of the induction of logical rules for functional description

Second part of the rule generation process is related to the filtering procedure. Here, the user can choose if standard, *Q* *C* *o* *m* *p* *o* *u* *n* *d* (3) rule interestingness measure should be used to rank the rules or the user can influence the process of rule interestingess assessment. In the latter case, the user can either decide on particular elements of the *Q* *C* *o* *m* *p* *o* *u* *n* *d* measure that it should include or decide to compute the complex additive *Q*_*UTA*_ measure which is then used to rank the rules. Then, as described earlier, based on the ranking, filtering procedure can be performed. The whole rule induction workflow is presented in the Fig. 1.

## Results and discussion

In this study, we propose a comprehensive framework for the generation of logical rules for functional description of gene sets using the controlled vocabulary from Gene Ontology database. We also present a new method for logical rule generation which allows the expert to verify hypothesis on existence or co-existence of specific pathways that are related to the experimental conditions.

To demonstrate how the method works, and compare different approaches to rule induction, we analyze gene signature from DNA microarray experiments that differentiate among three sub-types of breast cancer [48]. We use this dataset as a case study to show the proposed framework and its applications. The gene signature consists of 26 genes, and we assume that those genes should be involved in some processes related to tumor development. There is also reference set of 135 genes that are differentially expressed during the experiment. The lists of genes analyzed in this research are also provided in the Additional file 1.

For the breast cancer dataset, we generated logical rules using the four approaches presented earlier and compared their performance and accuracy. For the rule induction method with expert terms, we also analyzed how its composition changes depending on the selection of different components of *Q* *C* *o* *m* *p* *o* *u* *n* *d* measure and different filtering settings.

As the seed terms, we decided to choose the set of Gene Ontology terms that are related to the so-called hallmarks of cancer [49], that is cell capabilities that enable tumor growth and metastatic dissemination. The list of such GO terms related to hallmarks of cancer was derived from the Knijnenburg *et al* [50], in which they propose mappings from cancer hallmarks to Gene Ontology processes. Initial mapping included 57 GO terms, divided into 10 categories related to the following processes:*tissue invasion and metastasis, genome instability, tumor-promoting inflammation, reprogramming energy metabolism and evading immune destruction*, *sustaining proliferative signaling, evading growth suppressors, resisting cell death, replicative immortality*, and *sustained angiogenesis* (see Additional file 2 for the detailed list of GO terms). The set of 57 GO terms was extended with their children processes.

For the gene annotations, we used the version of Gene Ontology database from January 2016, GO terms from Biological Process only. The following settings for GO terms and the rule induction algorithm were applied:

- minimal level on GO graph: 3,
- maximal level on GO graph: 20,
- minimal number of genes described by a single GO term: 3,
- take into account GO graph hierarchy during analyses: yes,
- statistical significance level: 0.05,
- minimal rule support: 3,
- maximal number of GO terms in rule premise: 5,
- rule similarity threshold during filtering: 0.5.

After applying the above constrains on GO terms used during gene annotation process, we obtained 927 GO terms describing 134 genes both from analyzed (22 genes) and reference (112 genes) sets.

Obtained rule sets and their characteristics for different approaches for rule generation are presented in Table 1. Each column represents different approaches to rule generation according to the framework presented in this paper. Set S01 represents the results for the "raw" RuleGO method without filtering procedure applied, set S02 is a standard method with applied filtering, set S03 applies filtering using UTA approach, and set S04 proposes the new rule generation method that allows the user to control the process of logical rule generation by providing a set of seed terms. For the last approach, we also analyze how different *Q* *C* *o* *m* *p* *o* *u* *n* *d* measure and filtering parameters setting can influence the rule induction process. These results are presented in Table 1 as S04(1)-S04(6) datasets. Numbers from 1 to 6 in parenthesis after the name of the set S04 denote different parameter settings of rule quality assessment and filtering. The detailed information about the parameters setting for all sets is presented in Table 2. YES means that the particular component (*mYAILS*/*Lenght*/ *G* *O* *D* *e* *p* *t* *h*) of the *Q* *C* *o* *m* *p* *o* *u* *n* *d* measure or particular step of filtering procedure is the number of unique GO terms that was applied during the rule generation, NO means that the component was removed from the formula, in case of *Q* *C* *o* *m* *p* *o* *u* *n* *d* measure, or was not applied, in case of filtering.

**Table 1:** Comparison of different logical rule generation methods and different parameter settings

	S01	S02	S03	S04(1)	S04(2)	S04(3)	S04(4)	S04(5)	S04(6)
No. of rules before filtering	3812	3812	3812	110	110	110	110	110	110
No. of output rules	3812	32	32	9	10	7	19	110	14
No. of rules with expert terms	1465	15	11	9	10	7	19	110	14
Coverage	**82**	**82**	**82**	64	64	64	64	64	64
Avg. p-value	0.018	0.017	0.014	**0.009**	0.012	0.013	0.019	0.016	0.014
Avg. precision	0.74	0.78	0.77	**0.81**	0.78	0.7	0.68	0.71	0.72
Avg. coverage	0.14	0.15	0.15	0.16	0.16	0.16	0.16	0.15	**0.17**
Avg. GO Level	4.06	4.18	3.7	4.95	4.84	**5.8**	4.66	4.51	4.7
Positive coverage	**18**	**18**	**18**	14	14	14	14	14	14
Negative coverage	57	35	36	**11**	12	13	19	20	14
Positive coverage - expert rules	**14**	13	11	**14**	**14**	**14**	**14**	**14**	**14**
Negative coverage - expert rules	28	10	**11**	**11**	12	13	19	20	14
Avg. no. of descriptors	**3.57**	3.19	3.53	2.33	2.5	1.43	2.47	2.66	2.36
Avg. no. of expert term per rule	0.41	0.47	0.38	**1.44**	1.4	1.14	1.53	1.35	1.29
Number of distinctive expert terms	**19**	8	6	9	9	8	13	**19**	11

S01 – RuleGO method without filtering procedure, S02 – standard RuleGO method with applied filtering, S03 – filtering using UTA approach, S04 – new rule generation approach using seed terms. Description of different Q Compound measure and filtering setting for S04(1)-S04(6) is presented in Table 2

**Table 2:** List of parameters used for different rule induction methods as presented in Table 1

	Dataset	S01	S02	S03	S04(1)	S04(2)	S04(3)	S04(4)	S04(5)	S04(6)
	mYAILS	YES	YES	YES	YES	**NO**	**NO**	**NO**	**NO**	**NO**
QCompound	Length	YES	YES	YES	YES	YES	**NO**	**NO**	**NO**	YES
	GO_Depth	YES	YES	YES	YES	YES	YES	YES	YES	YES
Filtering	1st level	YES	YES	YES	YES	YES	YES	YES	**NO**	YES
	2st level	YES	YES	YES	YES	YES	YES	**NO**	**NO**	**NO**

YES means that the particular component of the Q Compound measure or particular step of filtering procedure was applied during the rule generation, NO means that the component was removed from the formula, in case of Q Compound measure, or, in case of filtering, was not applied. Columns represent different approaches to rule induction process and are consistent with the description of columns in Table 1

The analysis of the results presented in Table 1 shows that with the proposed new method the expert is able to obtain the description that includes terms that could be possibly interesting regarding the experimental conditions. The terms defined by the expert are combined with other GO terms providing the information on gene/protein functions. In case of no filtering, the number of rules is too large to be analyzed by human. Filtering allows reducing the number of rules, but in comparison with the new method, the output rule set is generated in a fully automated way and therefore consists of significantly less rules including expert GO terms. It is worth to notice that the method is designed in such a way that the filtering process does not reduce the coverage of gene set, which means that the algorithm always provides functional description for all genes that could be described by a set of GO terms satisfying constrains defined by the user. The difference in coverage between the automated and the expert-driven approach is the result of the fact that in case of the expert-driven procedure, we require each rule to have at least one expert GO term in its premise, and the maximal coverage of the gene set with provided expert terms is 62%. In case the user would like to obtain the description for the rest of genes from the group, the solution is to generate rules for the remaining genes in an automated manner.

Regardless of the rule induction method, after filtering, the average precision and the average coverage of the rules in the output set is higher. Thus, filtering allows to reduce some of rules from the output set which are too general, that is, describe not only genes from the analyzed set but also from the reference set, and it also prefers the rules that are supported by large number of genes (that is, describe more genes from the output rule set).

Analyzing the results from the UTA experiment (rule set S03), we can see that the rule ranking obtained with the *Q*_*UTA*_ measure, which is the base for the filtering process, allows generating final rule set that is characterized by similar values of quality indices as in the case of the rules sorted by the *Q* *C* *o* *m* *p* *o* *u* *n* *d* measure. This indicates that the method for semi-random sampling of rules which is used in the UTA approach allows selecting small subset of good representatives. This is important to notice, as the number of rules presented to the expert should be small enough for him or her to analyze them.

The UTA method takes into account the expert preferences, but in a different way than does the method based on seed terms. In case of the UTA method, a small set of representative rules is presented to the expert and he or she orders the rules according to his or her preferences. In other words, the expert shows the best way to order the rules, and the algorithm uses several objective indices to reconstruct the ranking. In this approach, it is difficult to provide the exact definition of the criteria on which the rules are ordered, as it is more related to the expertise and preferences of a particular person. Therefore, we may see it as the *soft* approach to expert-driven rule induction process.

As the opposite to the UTA expert-driven rule generation algorithm, the method based on seed terms can be seen as a *hard* approach to rule induction and filtering. Here, the user decides which GO terms are interesting for him or her and each generated rule must include at least one of the GO terms from the expert set. The simplest possible description that includes GO terms that are interesting from the expert point of view is represented in Table 1 as the results set S04(1). We can notice that the number of output rules in this set is very small, and this because the rules that are added to the output rule set are selected restrictively. However, they are characterized (in average) by the smallest *p*-value, highest precision, very high coverage, and the smallest coverage of the negative class. Also in this set the average number of expert GO terms per rule is the highest among all rule set generated in this study. This may be interpreted as the fact that the provided set of expert terms is functionally related to the analyzed gene signature.

Analyses of the other results for the set S04, that is, results obtained with different sets of parameters for *Q* *C* *o* *m* *p* *o* *u* *n* *d* measure and different filtering settings, show that the user is able control the process of rule induction. The proposed system is designed in a flexible way, and the user can influence the process of rule induction not only by indicating the biological processes of special interest but also by having the possibility to decide on the characteristics of the output rule set by influencing the process of rules evaluation and thus rules filtering.

**Fig. 2:**
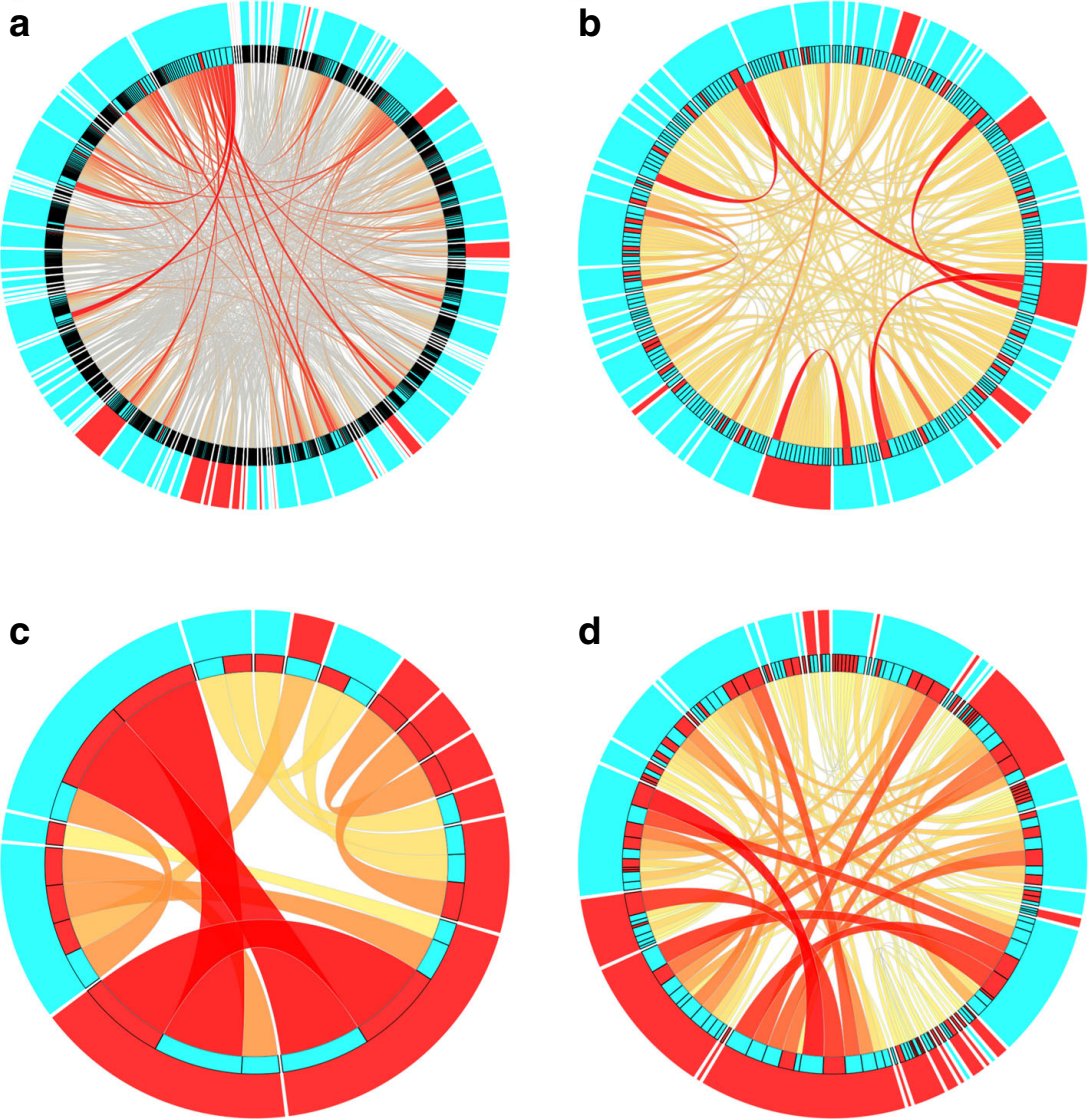
Visualization of rule networks obtained for the selected rule induction methods. Each circle represents rule network obtained by using different methods. **a** – rule network set S01, **b** – rule network set S02, **c** – rule network set S04(1), **d** – rule network set S04(5)

To show the proportion of expert terms in rule sets generated by using different methods, we also visualize the composition of expert terms in the output rules. The visualization is presented in Fig. 2. In order to do this, we used ciruvis tool [51], which allows visualizing of rule networks. On the presented visualization, GO terms are represented as outer and inner edges of the circle, and the connections among the GO terms are shown as edges between the elements. The inner ring shows the color of the GO terms on the other side of the connection. Here, the expert GO terms are represented by red color and all the other GO terms describing gene set are represented in blue color.

Analyzing the information presented in the graphical form in Fig. 2, we can notice that in case of no filtering step (Fig. 2 a), even if the number of expert terms is the biggest compared with other results, there is also a lot of additional information that makes it a difficult task to find the particular processes in the huge output rule set. Then, we can observe that filtering (Fig. 2 b) is able to reduce the number of rules; however, it also removes the rules including expert GO terms from the results. In case of the new, expert-driven rule induction and filtering method (Fig. 2 c and d), we see that the expert terms are substantial part of the output rule set. By applying the filtering process with different sets of parameters, the user can decide how much additional information should be included into the final description. Rule network sets used to generate Fig. 2 are provided in Additional file 3.

## Conclusion

In this study, we presented the comprehensive framework for logical rule induction for functional interpretation of the results of high throughput experiments. In order to obtain the description, we use controlled vocabulary from the Gene Ontology database as the keywords that help the expert to understand and interpret the results of experiments by means of the so-called logical rules in the form of combinations of GO terms.

Based on the results, we recommend that in case when there is no specific domain knowledge related to the experimental data or conditions, the user should use standard RuleGO rule generation method with the filtering procedure. In case when the expert want to be involved in the process of rule generation, but still is not focused on particular processes and pathways, the UTA method for filtering the rules should be used. However, in case when domain knowledge related to the experimental data exists, and if the expert prefers to influence the process of rule induction or verify the hypothesis on existence of particular pathways, we propose the rule generation process in which the rules are generated based on the expert terms.

In this work, we presented and compared four basic approaches to the generation of rules for description purposes, including a new method for rule generation based on expert terms. We showed that the filtering step is needed to reduce the output set, so that it could be analyzed by the human expert. Then we presented two methods that involve the interaction with the expert during the process of rule induction. Both of them are able to reduce the number of rules, but only in the case of the method based on seed terms, each of the created rule includes expert terms in combination with the other terms. Further analysis of such combinations may provide new knowledge about the biological processes and their combination with other pathways related to genes described by the rules.

A suite of Matlab scripts that provide the functionality of a comprehensive framework for the rule induction and filtering presented in this study is available free of charge at: http://rulego.polsl.pl/framework.

## Additional files

Additional file 1 Analyzed gene set and reference gene set. This excel file includes 26 genes that compose described gene group *G*_1_ and 135 genes from reference gene group *G*_2_ analyzed in this study. The lists of genes were derived from Finak et al. [48]. (XLSX 33 kb)

Additional file 2 List of 57 GO terms related to hallmarks of cancer. This excel file includes a list of 57 GO terms related to the hallmarks of cancer from Knijnenburg et al. [50], which were used as a base to define the expert terms used in the analysis. (XLSX 29 kb)

Additional file 3 Rule network sets that were generated as a result of this analysis. This excel file includes rule network sets that were generated as a result of this analysis. Each tab of the Excel file represents different rule set: set S01 represents results for the RuleGO method without filtering procedure applied, set S02 is a standard method with applied filtering, set S03 applies filtering using UTA approach, and set S04 proposes a new approach that allows the user to control the process of logical rule generation by providing a set of seed terms. For the set S04, we provide six rule sets obtained with different sets of quality assessment and filtering parameters as presented in Table 2. Each row represents a single rule, and GO terms from the expert set (seed terms) are denoted with star (*). Also for sets S02, S03, and S04 seed terms are marked with bold font. For each rule, we also provide the number of genes supported and recognized by the rule, its precision, coverage, value of the *QCompound* measure, *p*-value, and the list of genes supported by that rule. (XLSX 257 kb)
